# Identification of biomarkers based on ubiquitin-correlated genes for predicting immune profile and drug sensitivity in lung adenocarcinoma

**DOI:** 10.3389/fphar.2025.1646396

**Published:** 2025-08-15

**Authors:** Yue Li, Wei Tian, Chen Chen, Hailin Liu, Zhenfa Zhang, Changli Wang

**Affiliations:** ^1^ Department of Lung Cancer, Key Laboratory of Cancer Prevention and Therapy, Tianjin’s Clinical Research Center for Cancer, Tianjin Lung Cancer Center, National Clinical Research Center for Cancer, Tianjin Medical University Cancer Institute and Hospital, Tianjin, China; ^2^ Department of General Surgery, The Second Affiliated Hospital of Tianjin University of Traditional Chinese Medicine, Tianjin, China

**Keywords:** lung adenocarcinoma, ubiquitin, prognostic modeling, immune characterization, drug sensitivity

## Abstract

**Background:**

Lung adenocarcinoma (LUAD) shows high recurrence rate and poor prognosis. Genes associated with ubiquitin play a role in the onset and advancement of cancers; however, they have yet to be employed for the diagnosis and prognosis of LUAD.

**Methods:**

First, gene modules correlated with ubiquitin were identified by WGCNA. The expression profiles obtained were intersected with differential genes taken between the LUAD and control samples. The genes were then further compressed using univariate and multifactorial Cox regression analyses and risk models. In addition, the model was validated by constructing a nomogram using clinical characteristics and Riskscore. Next, the differences in immune infiltration between different subgroups were explored, and immunotherapy and drug sensitivity evaluations were performed. The biological role of *HEATR1* in LUAD was also explored using CCK-8, wound healing assay and transwell.

**Results:**

The intersection between the module genes between LUAD samples and control samples and differentially expressed genes (DEGs) yielded 197 intersected genes after we screened three particular modules with the strongest ubiquitin association by WGCNA. 32 genes associated with LUAD prognosis were screened, and *B4GALT4*, *DNAJB4*, *GORAB*, *HEATR1*, *LPGAT1*, *FAT1*, *GAB2*, *MTMR4* and *TCP11L2* were selected as independent prognosis genes for risk modeling. Patients were classified into low- and high-risk groups by the Riskscore. Low-risk patients had markedly better overall survival (OS) than those in the high-risk group. The quantity of immune cell infiltration between the two patient groups varied notably, and the expression of model genes was negatively connected with the infiltration of the great majority of immune cells. The medications TAE684, Cisplatin, and Midostaurin exhibited the largest negative correlation with Riskscore, according to drug sensitivity study. Lastly, we demonstrated through *in vitro* tests that *HEATR1* knockdown markedly reduced LUAD cell survival, migration, and invasion.

**Conclusion:**

This study is the first to systematically integrate the ubiquitin pathway with multi-omics data, constructing a robust risk model for LUAD prognosis and immune characteristics, providing a theoretical reference for future exploration of potential biomarkers for LUAD patients' diagnosis.

## Introduction

Around 50% of all lung malignancies are lung adenocarcinoma (LUAD), which is the primary histologic subtype of lung cancer and a major cause of cancer-correlated mortality worldwide (Sung et al., 2021; Little et al., 2007). In recent decades, considerable advancements have been achieved in understanding the molecular mechanisms underlying LUAD progression, resulting in the creation of targeted therapies like tyrosine kinase inhibitors (Chaft et al., 2021). In addition, new treatment strategies have been proposed, particularly immunotherapy (including immune checkpoint inhibitors and adoptive cell therapy), which show promising results for patients whose cancer has spread and who no longer respond to traditional treatments (Ren et al., 2023). Nevertheless, only a small number of patients benefited from immunotherapy, highlighting the urgent need to identify potential biomarkers for effective prognostic predictions.

Since post-translational modification can precisely regulate protein function, thereby determining cellular metabolism or responses to genetic and environmental changes, this process is crucial (Cappadocia and Lima, 2018). Ubiquitin-mediated post-translational alterations can alter the level, location, and function of its particular targets (Clague et al., 2015). Through an enzymatic cascade process involving three enzymes—E1 (activating enzyme), E2 (conjugating enzyme), and E3 (ligase)—these modifiers covalently bond to their targets. The E1 enzyme activates ubiquitin and creates a covalent thioester link by catalyzing the adenylation of its C-terminal end with the help of adenosine triphosphate. The active ubiquitin is subsequently taken up by the E2 enzyme via a transsulfurylation process. Lastly, ubiquitin is transferred to particular target lysine residues by the ubiquitin-containing E2 enzyme, either by itself or in conjunction with the chaperone E3 enzyme, creating an amide (or isopeptide) bond between ubiquitin and the substrate. Ubiquitin dissociation, editing, and recycling are carried out by deubiquitinating enzymes and ubiquitin-specific proteases (Guo et al., 2023; Hwang et al., 2022). It has recently been shown that a number of ubiquitins are intimately associated with the development and metastasis of different forms of cancer. The deubiquitinating enzyme USP7, for instance, promotes the progression of colorectal cancer by up-regulating several cellular pathways, such as Wnt/β-catenin signaling (Zhao et al., 2024; Li X. et al., 2020). According to Liao et al. (Liao et al., 2021), the deubiquitinating enzyme USP1 is responsible for the advancement of prostate cancer by blocking the K48-linked polyubiquitination of SIX1. Upregulating the ubiquitin-specific peptidase USP48 reduces hepatocellular cancer by changing SIRT6 stabilization (Du et al., 2021). In breast cancer, hyperactivation of the deubiquitinase USP1 promotes metastasis of breast cancer cells to lung, upregulates the expressions of many pro-metastatic genes in cancer cells, and enhances cell migration and invasion *in vitro* (Ma et al., 2019). Nevertheless, nothing is known about the expression status, activity, and role of ubiquitin in LUAD. Thus, the hunt for ubiquitin-related indicators in LUAD might yield fresh concepts for cancer treatment.

This research used bioinformatics to examine the ubiquitin-related gene scores of LUAD patients in public databases. WGCNA was used to identify and enhance the modular genes linked to ubiquitin in LUAD. A Riskscore system was developed using the screened characteristic genes linked to the prognosis of LUAD. LUAD patients were divided by the median Riskscore into low-risk and high-risk groups, and a nomogram of the Riskscore in relation to the various clinicopathological characteristics of LUAD patients was created. The correlation of the risk model with drug sensitization and immunotherapy was analyzed. Overall, our study provides a new method to predict the prognosis and immune infiltration of LUAD patients based on ubiquitin-related genes, which may provide new insights into prognostic assessment and therapeutic strategies for LUAD.

## Methods

### Data acquisition and preprocessing

The Cancer Genome Atlas (TCGA) database (https://portal.gdc.cancer.gov/) was accessed to collect clinical information of LUAD and gene expression profiles. After removing samples without survival time or status, the included patients had a survival time longer than 0 days. RNA-seq expression profiles were collected and transformed to reads per million mapped (FPKM) format and log2 transformed, resulting in 500 lung cancer samples and 59 control samples for final screening. Microarray data for GSE31210 were downloaded from Gene Expression Omnibus (GEO, https://www.ncbi.nlm.nih.gov/geo/) database. Based on the annotation file, normal tissue samples were removed by converting the probe to Symbol. Samples without overall survival (OS) or clinical follow-up data were subsequently removed, and a sum of 226 tumor samples were finally screened by GSE31210. This study used TCGA-LUAD as the training set and the GSE31210 dataset as the independent validation set. Subsequently, the iUUCD 2.0 database (http://iuucd.biocuckoo.org/) was accessed to obtain a total of 807 ubiquitin-related genes (URGs). The URGs scores for each sample in TCGA_LUAD cohort were calculated using single-sample gene set enrichment analysis (ssGSEA) in the “GSVA” package (Subramanian et al., 2005).

### Weighted gene co-expression network construction

WGCNA was then used to preprocess the gene expression data, eliminating genes with low variance and keeping only those with high variance (Wang Y. et al., 2024). In particular, we screened the genes using the mad (median absolute deviation) measure, set a threshold of 0.01 and chose the top 75% of genes with higher variance. Then, the proper soft threshold β was found using the pickSoftThreshold function. Once the soft threshold was established, the topological reconstruction similarity (TOM) between genes was further computed using a weighted neighbor-joining matrix. The genes were then grouped using the hierarchical clustering approach, and the topological reconstruction dissimilarity (dissTOM) was used to calculate the distance between the genes. The minimal number of module genes was set at 60 during the module identification procedure, with deepSplit = 2, and the cut height for module merging was set at mergeCutHeight = 0.3. We computed and grouped each module’s feature gene vectors in order to examine the connection between gene modules and URGs feature scores in more detail. Based on this, the “Heatmap” package (Saunders et al., 2007) was used to conduct module-trait correlation analysis. Pearson correlation coefficients between each module and URGs trait scores were then obtained, and the corPvalueStudent function was used to determine the appropriate *p*-values. To assist in identifying key modules associated with URGs function, we use heatmaps to illustrate the relationship between modules and URGs feature scores. The genes contained in the modules were filtered out of the modules having the highest correlations. Finally, we computed Gene Module Membership for module genes and Gene Trait Significance for each gene with respect to URGs trait scores in order to obtain a better understanding of the characterization of genes within a module. We then used scatter plots to illustrate the connection between module membership and gene trait significance.

### Gene enrichment analysis

Using the “clusterProfiler” package (Yu et al., 2012), the modular genes derived from WGCNA screening were subjected to Gene Ontology (GO) and Kyoto Encyclopedia of Genes and Genomes (KEGG) enrichment analyses, with *p* < 0.05 considered statistically significant (Wang et al., 2025). The Top 10 GO terms (for BP, MF, and CC) and KEGG pathways were selected based on gene count and adjusted p-values, and visualized using bubble plots to highlight the most relevant biological functions.

### Screening of differentially expressed genes

The “limma” package (Ritchie et al., 2015) was employed to select DEGs between LUAD cases and control samples in the TCGA cohort. Using *p*.value < 0.05 and |log2 (FC)| > log2 (1.5) as statistical significance criteria, gene expression profiling data were background adjusted, quartile normalized, and professionally summarized to screen for significant DEGs. The junction of modular genes and DEGs derived from the WGCNA screen was then captured.

### Risk modeling and validation

We next performed univariate Cox proportional risk regression on intersecting genes using the R package “survival” (Lirette and Aban, 2017) to identify genes closely linked to the prognosis of patients in the TCGA_LUAD cohort. Multifactorial stepwise regression analysis was then used to develop a Riskscore model, which screened the TCGA_LUAD dataset for significant genes and correlation coefficients that were independently associated with patient prognosis. The following formula was used to determine the Riskscore: Riskscore = Σβi × Expi. Expi is the expression of every gene gathered, i denotes the gene expression level, and β is the associated gene’s Cox regression coefficient. Following zscore normalization, the Riskscore’s ideal critical value was used to divide the TCGA_LUAD dataset’s patients into low-risk and high-risk groups. Plotting Kaplan-Meier (K-M) survival curves and performing survival analyses comparing the two risk groups using the R package “survminer” (Ozhan et al., 2021) were then used to conduct prognostic studies. Additionally, we calculated the AUC for the TCGA-LUAD training and test sets at 1-, 2-, 3-, 4-, and 5-year AUC and assessed the predictive model’s performance by the ROC curves using the R package “timeROC” (Blanche et al., 2013) in order to evaluate the diagnostic accuracy of the prognostic risk model. Finally, we applied the same techniques to the GSE31210 dataset to further validate the reliability and stability of our established clinical prognostic models based on risk-related gene signatures.

### Establishment and validation of prognostic nomogram

Univariate and multivariate Cox regression analyses combining the various clinical variables of LUAD (age, gender, T.stage, N.stage, M.stage, stage) were utilized in order to ascertain whether Riskscore was an independent prognostic factor predicting the survival of LUAD patients. The clinical characteristics identified by the prognostic model and multivariate regression analysis were then combined to create a prediction nomogram (Iasonos et al., 2008). To assess the nomogram’s deviation from the ideal model, the “caret” package (Kuhn, 2008) produced calibration curves, and the predictive ability of the model was assessed by using the “rmda” package (Gerds et al., 2014) in R software to plot decision curve analysis (DCA).

### Immune infiltration analysis

To examine the relationship between Riskscore and LUAD immune function, the immune cell scores of the TCGA dataset in different subgroups were evaluated using the tumor immunity estimate resource (TIMER) database (https://cistrome.shinyapps.io/timer/) (Li et al., 2017). Furthermore, the correlation between the Riskscore and the 10 immune cell ratings of the TCGA-LUAD dataset was ascertained using the “MCPcounter” package (Becht et al., 2016). Based on the transcriptome expression profiles of the samples, we then investigated the correlation between the hub genes and the infiltration patterns of the 28 immune cells (Charoentong et al., 2017) using the ssGSEA function of the “GSVA” package for the scores of the TCGA-LUAD cohort.

### Immunotherapy relevance and drug sensitivity analysis

TIDE describes T-cell malfunction and influences patient survival and immunotherapy response by utilizing the correlation between the tumor’s gene expression profiles and the extent of cytotoxic T-lymphocyte infiltration (Fu et al., 2020). Patients with a higher TIDE prediction score have higher immune escape potential, which implies that immunotherapy has a lower chance of success and less likely to benefit in any way. Using the TIDE (http://tide.dfci.harvard.edu/) database, we first evaluated the possible clinical effects of immunotherapy in low- and high-risk groups in order to ascertain the significance of genes to the advantages of immunotherapy. Then, we used the “pRRophetic” package (Geeleher et al., 2014) in R to analyze the sensitivities of commonly used chemotherapeutic drugs in the TCGA-LUAD cohort and to determine the correlation between the half-maximal inhibitory concentration (IC_50_) and the Riskscore, with *p* < 0.05 deemed significant. Spearman correlation analysis was employed to evaluate the relationship between the predicted IC_50_ values and the Riskscore across samples.

### Cell culture and plasmid transfection

Human normal lung epithelial cells (BEAS-2B, CRL-3588) and human LUAD cells (H2228, CRL-5935 and A549, CRM-CCL-185) purchased from the American Type Culture Collection were cultivated in RPMI-1640 medium (11875093, Gibco, United States) containing 10% fetal bovine serum (FBS, 10099141, Gibco, United States) and 100 U/mL of penicillin and streptomycin (15140122, Gibco, United States). Every cell was cultivated in an incubator at 37 °C with 5% CO_2_. We have performed STR identification on the cells, and the *mycoplasma* detection results turned out to be negative.

*HEATR1* knockdown plasmid (si-*HEATR1*) and control plasmid (si-NC) ordered from GenePharma (Shanghai, China) were transfected into 2 × 10^4^ cells/well of H2228 and A549 cells, respectively, according to the instructions of Lipo3000 Liposomal Transfection Reagent (L3000-001, ThermoScientific, Waltham, MA, United States). si-*HEATR1* sequence was as follows: 5′-UGG​AUU​GUA​CCA​UUC​UUC​UG-3' (si-*HEATR1*#1) and 5′- TCC​TTC​CTT​TGA​GCA​GTT​TGA​AG-3’ (si-*HEATR1*#2).

### RNA extraction and quantitative real-time PCR

Total RNA from BEAS-2B, H2228, and A549 cells was isolated utilizing the RNA Extraction Kit (TRIzol, Invitrogen, United States) in accordance with the instructions. The concentration and purity of the total RNA extracted were tested. The creation of cDNA templates was then initiated using the HiScript II kit (Vazyme, China). Specific primers and the KAPA SYBR^®^ FAST kit (Sigma Aldrich, San Luis, MO, United States) were used for qRT-PCR. The conditions for the PCR reaction are outlined as follows: 30 s at 95 °C, followed by 10 s at 95 °C, then 30 s at 60 °C, and finally 34 s at 70 °C, totaling 40 cycles. Relative gene expression was computed employing the 2^−ΔΔCT^ technique was utilized to examine the data, while *GAPDH* as an internal control. Primer sequences for particular genes were shown in Table 1.

**TABLE 1:** The sequences of primers for RT−qPCR used in this study.

Gene name	Forward primer	Reverse primer
B4GALT4	5′ CTC​TGA​CTA​ATG​AAG​CAT​CCA​CG 3′	5′ CTG​CCT​GTA​CCT​CTT​CCA​AAG​TG 3′
DNAJB4	5′ TTA​AAG​AGG​TCG​CAG​AAG​CTT​ATG 3′	5′ GAT​CGC​CAT​GAA​AGG​TGT​ACC​G 3′
GORAB	5′ CCA​AAA​GAA​CTC​AGG​CAG​AGA​CC 3′	5′ CTT​CAG​CCC​TGT​CAA​ACC​GCT​T 3′
HEATR1	5′ GTC​CGA​ATA​GAA​CTG​GAG​CCA​C 3′	5′ GCC​AGT​AAG​AAC​CTC​CAA​CTT​CC 3′
LPGAT1	5′ TAC​CAC​TTG​GCT​CTA​TCA​GCG​G 3′	5′ CCA​CAA​GTT​GCT​GAG​GGT​CAT​C 3′
FAT1	5′ ATC​TGT​GGA​GCC​TCC​TGG​CAT​A 3′	5′ CAT​CTG​TAG​CCT​CGA​CTG​TGA​G 3′
GAB2	5′ TCA​GCA​GAG​ACC​GCC​AAT​CAG​T 3′	5′ GGT​ACT​CGT​AGG​TCT​CAC​AGG​A 3′
MTMR4	5′ CTG​TGT​TCC​TCC​AGT​GGC​TTG​A 3′	5′ TGC​CGT​AGA​GGC​AGG​AGT​ATG​T 3′
TCP11L2	5′ AAG​GCT​ACT​GGC​AAC​ATC​GTG​G 3′	5′ ACC​AAC​TGG​CGT​TGA​AGG​TGC​G 3′
GAPDH	5′ GTC​TCC​TCT​GAC​TTC​AAC​AGC​G 3′	5′ ACC​ACC​CTG​TTG​CTG​TAG​CCA​A 3′

### Cell viability

To evaluate the effect of *HEATR1* on H2228 and A549 cell viability, colorimetric assays were performed following the protocol, CCK-8 (DOJINDO, Japan). Briefly, cells (2000 cells/Well) were grown in 96-well microplates for 0, 24, 48, and 72 h. Cells were washed with PBS twice, then 100 mL of fresh medium and CCK-8 solution (10 μL) were supplemented to each well and incubated with 5% CO_2_ at 37 °C for 3 h. Using a microplate reader SPECTROstar Nano (BMG LABTECH GmbH, Ortenberg, Germany), the absorbance at 450 nm was determined.

### Cell migration and invasion assays

To assess the effect of *HEATR1* on the migration and invasion ability of H2228 and A549 cells, we subsequently performed scratch and transwell assays. In the invasion assay, H2228 and A549 cell suspensions (5 × 10^5^/mL) were prepared with serum-free medium. Then, 100 μL of cell suspension was filled into the upper transwell chamber (Corning, Beijing, China) pre-coated with Martigel (30 μg/well; BD, San Jose, CA, United States), while the lower chamber contained 600 μL of RPMI-1640 medium with10% FBS. Migrated cells were fixed with 4% paraformaldehyde and dyed with crystal violet solution. These migrated or invaded cells in the lower chamber were quantified under a microscope (CKX41; Olympus, Tokyo, Japan) using 6 different fields of view (Fan, 2023).

For migration assays, collective cell migration was detected by wound healing assay. Transfected cells were inoculated into 6-well plates (5 × 10^5^/mL). Two mL of cell suspension was inoculated into 6-well plates and incubated at 37 °C and 5% CO_2_ in an incubator. When the cells were adherent to the wall, the monolayer was scraped with a 10 μL plastic pipette tip to form a uniform wound. After washing with PBS, the monolayers were incubated in non-FBS medium. The wound edge distances between the two edges of the migrating cell sheet were photographed at 0 h and 48 h, respectively. All the experiments were conducted in triplicate (Zh et al., 2024).

### Statistical tests

GraphPad Prism 8 (GraphPad Software, San Diego, United States) and R software version 3.6.0 (R Foundation, Vienna, Austria) were employed in all statistical analyses. Kaplan-Meier (KM) survival curves and log-rank tests were used to compare survival differences between high- and low-risk groups. Spearman’s algorithm was used to analyze correlations. For cell experiments, Student's t-test was used to compare differences between two groups, or one-way ANOVA was used to test differences between multiple groups. All cell experiments were performed in triplicate. A *p*-value < 0.05 stood for statistical significance in all analyses.

## Result

### WGCNA screening for gene modules associated with URGs

**FIGURE 1:**
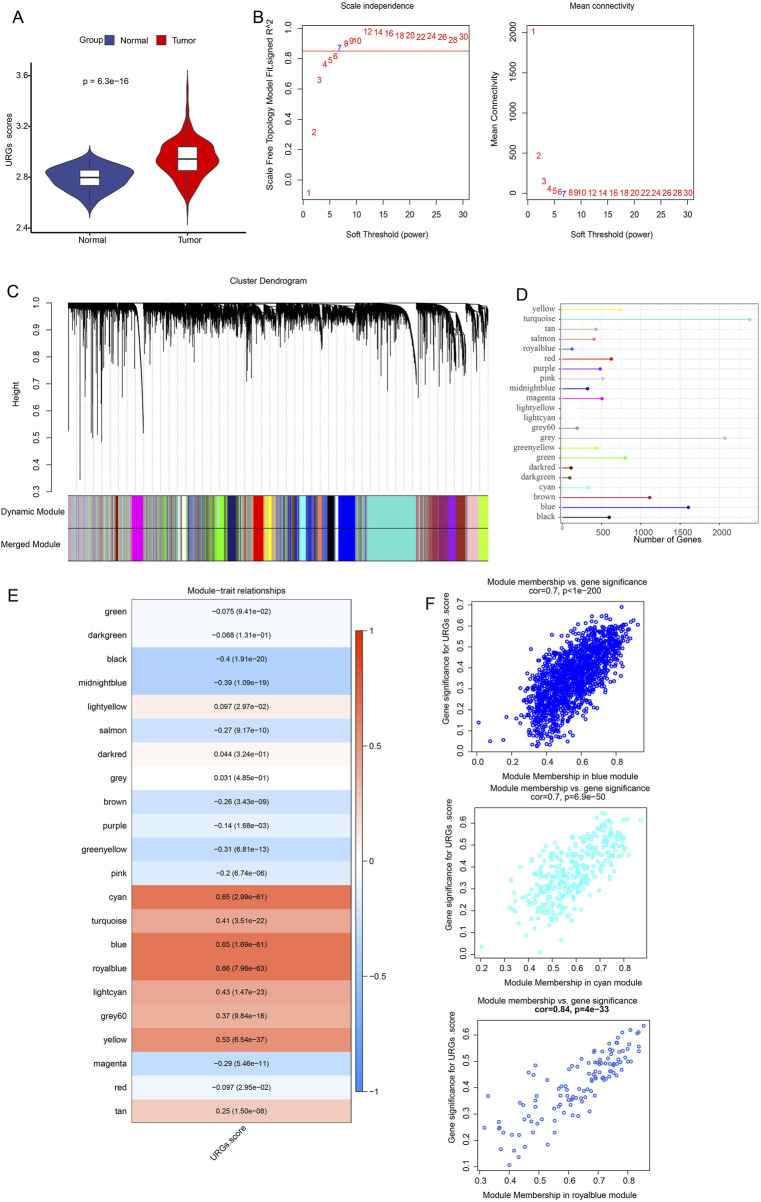
Identification of URGs-related gene modules in the TCGA dataset using WGCNA. **(A)** URG scores were obtained from ssGSEA calculations between normal and tumor. The scale-free fit index and average connectivity analyses of different soft-threshold powers (β) are presented in **(B)**. **(C)** Gene dendrogram based on clustering using the dissimilarity measure (1-TOM). **(D)** Each module’s gene count. **(E)** Heatmap showing the correlation between gene modules and URGs scores. Each cell contains the correlation coefficient and corresponding p-value. The depth of color represents the strength of the correlation between the module and the URGs score. Red indicates a positive correlation, blue indicates a negative correlation, and the deeper the color, the more significant the correlation. **(F)** Scatterplot of correlation between module membership degree and URGs score of module genes for cyan, blue and royalblue module genes.

First, we used the ssGSEA technique to evaluate the URG scores of each LUAD sample and control sample in the TCGA dataset. It was discovered that the tumor group’s URG score was noticeably greater than the control group’s (Figure 1A). URGs were thought to be connected to the development of LUAD. In order to find the gene modules associated with URGs in the TCGA dataset, the R package “WGCNA” was further used. After clustering the samples to screen the co-expression modules, we constructed the topological network using a soft threshold of β = 7 to guarantee that the network was scale-free (Figure 1B). Hierarchical clustering was then used to identify the gene modules. After the modules were merged, 22 co-expression modules were produced (Figure 1C), with the gray module representing the gene that was unable to be grouped into any other modules. Out of the 22 modules, the turquoise module had the comparatively most genes, followed by the grey and blue modules, as illustrated in Figure 1D. We computed each module’s connection with URG scores and created a module-shape correlation heat map in order to identify clinically significant modules. It can be seen that the cyan (cor = 0.65, *p* = 2.99e-61), blue (cor = 0.65, *p* = 1.69e-61) and royalblue (cor = 0.66, *p* = 7.96e-63) modules have a strong correlation with URGs scores were strongly correlated (Figure 1E). Therefore, we selected the cyan, blue and royalblue modules as clinically significant for further analysis. Lastly, we determined the Gene Module Membership of the module genes and the Gene Trait Significance of each gene in relation to the feature scores of the URGs in order to obtain a better understanding of the characterization of the genes inside the modules. The scatterplot indicates that the blue (cor = 0.7, *p* = 6.9e-50), royalblue (cor = 0.84, *p* = 4e-33), and cyan (cor = 0.7, *p* < 1e-200) module genes had a substantial positive association between module membership and URGs score (Figure 1F).

### Enrichment analysis of modular genes

**FIGURE 2:**
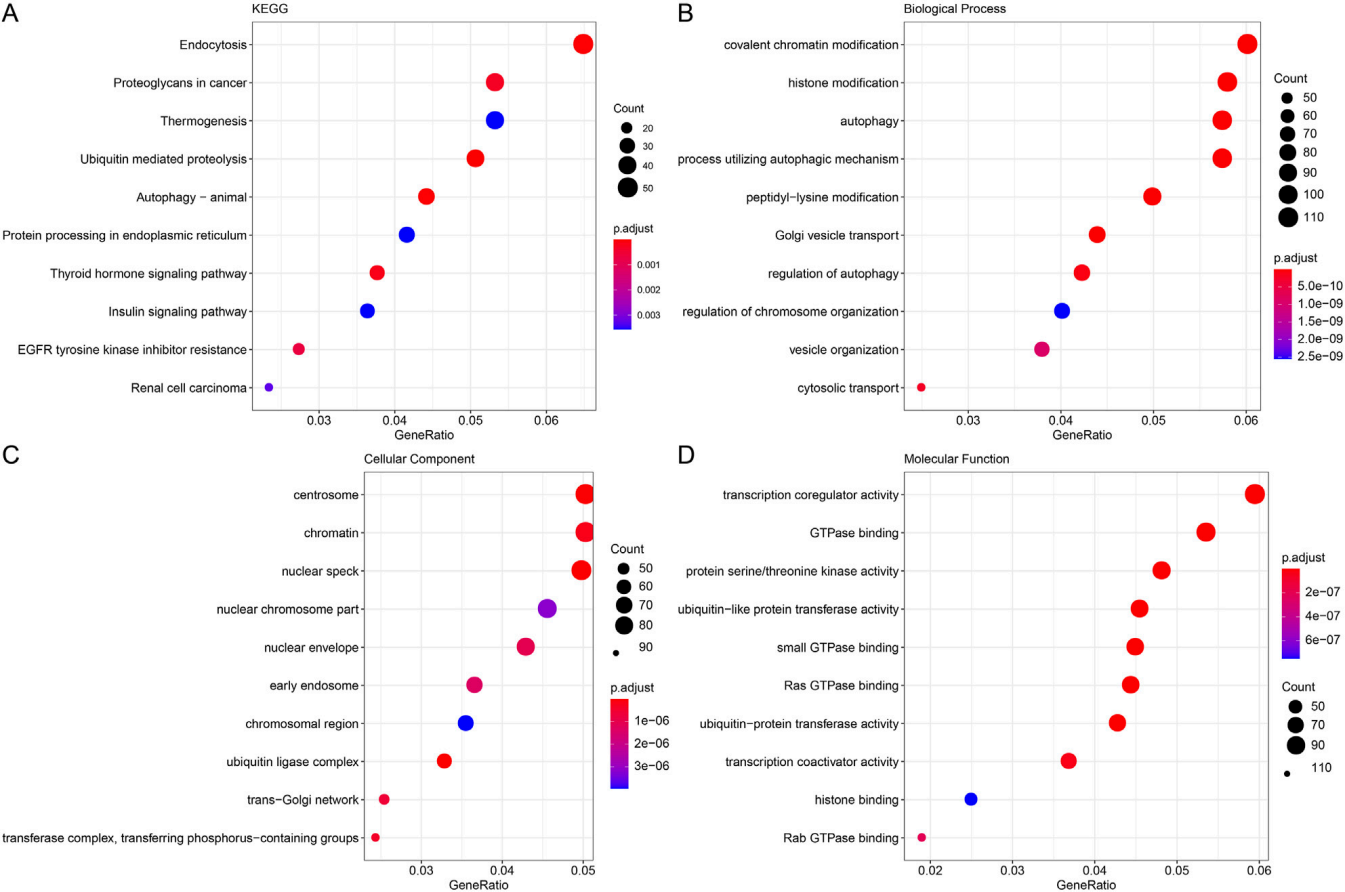
Functional enrichment analysis of modular genes. **(A)** Gene KEGG enrichment analysis bubble plot with horizontal coordinates representing the number of genes contained within the entries and colors representing the significance p-value, increasing in significance from blue to red. **(B)** Gene GO enrichment analysis BP bubble plot. **(C)** Gene GO enrichment analysis CC bubble plot. **(D)** Gene GO enrichment analysis MF bubble plot.

The genes in the modules were substantially enriched in endocytosis, ubiquitin proteolysis, and proteoglycans in cancer pathways, per KEGG analysis (Figure 2A). The modular genes were primarily involved in the BPs of covalent chromatin modification, chromatin modification, histone modification, according to GO enrichment analysis. Autophagy, histone modification, chromatin modification, and the use of autophagic mechanism pathways (Figure 2B). The centrosome, chromatin, nuclear speck, and other structures were the CCs where module genes were primarily enriched (Figure 2C). Pathways like transcription coregulator activity, GTPase interaction, and protein serine/threonine kinase activity were the MFs most significantly enriched for module genes (Figure 2D).

### Prognostic modeling and validation

**FIGURE 3:**
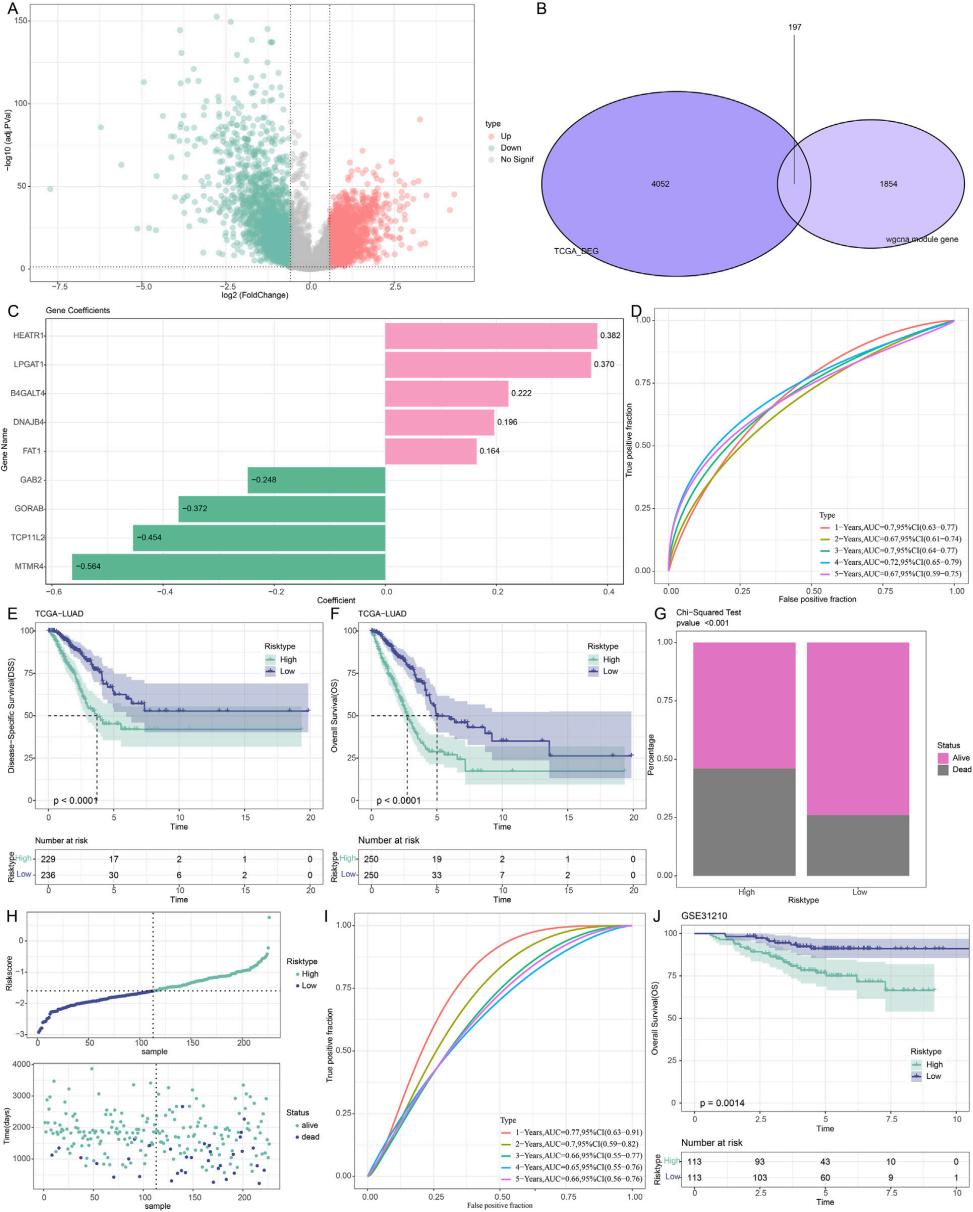
Development and verification of prognostic models for LUAD patients associated with URGs. **(A)** DEG volcano map of the TCGA cohort’s LUAD and control samples. **(B)** The junction of the cyan, blue, and royalblue module genes with differential genes. **(C)** The training set’s important genes’ risk coefficients. **(D–F)** TCGA dataset ROC curves, disease-specific survival (DSS) K-M curves, and OS K-M curves. **(G)** TCGA dataset risk modeling of survival status differences between high and low-risk groups. **(H)** Riskscore curves and survival status in the GSE31210 dataset. **(I,J)** ROC curves, OS K-M curves in the GSE31210 dataset.

The DEGs between LUAD and control samples were then found in the TCGA dataset. The volcano plot showing DEGs is displayed in Figure 3A. Additionally, we took the intersection of the DEGs with cyan, blue, and royalblue modular genes, which produced 197 URGs that were differentially expressed (Figure 3B). Subsequently, we used univariate Cox regression analysis in order to identify 32 genes that were strongly associated with survival in TCGA-LUAD patients (*p* < 0.05). Further using stepwise multifactorial Cox regression, nine (*B4GALT4*, *DNAJB4*, *GORAB*, *HEATR1*, *LPGAT1*, *FAT1*, *GAB2*, *MTMR4* and *TCP11L2*) genes independently associated with TCGA-LUAD prognosis were finally identified (Figure 3C). Subsequently, a characterization to assess the prognosis of TCGA-LUAD patients was developed based on the expression of the genes and the regression coefficients as described below: Riskscore = 0.222*B4GALT4+0.196*DNAJB4+(-0.372*GORAB) +0.382*HEATR1+ 0.37*LPGAT1+0.164* *FAT1* + (−0.248*GAB2) + (−0.564*MTMR4) + (−0.454*TCP11L2). According to the formula of the risk model, each sample Riskscore is calculated, and then the Riskscore is zscore standardized. According to the threshold “0”, the samples in the TCGA-LUAD training cohort were divided into high-risk and low-risk groups.

The AUC values in the TCGA-LUAD training set were 0.7, 0.67, 0.7, 0.72, and 0.67 at 1, 2-, 3-, 4- and 5 years, respectively, confirming the robustness of the prognostic characteristics in predicting OS in TCGA-LUAD patients (Figure 3D). Further analysis of the Riskscore and survival distribution in TCGA-LUAD patients revealed that the OS (*p* < 0.0001) and disease-specific survival (*p* < 0.0001) of the high-risk group were considerably worse than those of the low-risk group (Figures 3E,F). Upon comparing the two risk groups’ survival rates, we found that the high-risk group experienced a higher number of deaths (*p* < 0.001, Figure 3G). To verify the stability and reliability of our clinical prognostic models constructed using URGs, we employed the GSE31210 validation set to assess the prognostic models’ resilience using models and equivalence coefficients similar to those used for the identified training set. The validation results (Figure 3H) corroborated the training set finding that patients who died in the GSE31210 dataset were more concentrated in high-risk groups. The AUC values for the GSE31210 validation set were 0.77, 0.7, 0.66, 0.65, and 0.66 for the 1-, 2-, 3-, 4-, and 5-year periods, respectively (Figure 3I). In the GSE31210 validation cohort, patients in the high-risk group similarly performed worse (*p* < 0.0014, Figure 3J). Proving that model genes are accurate predictors of the prognosis of LUAD patients.

### Riskscore and key clinical features-based nomogram construction and validation

**FIGURE 4:**
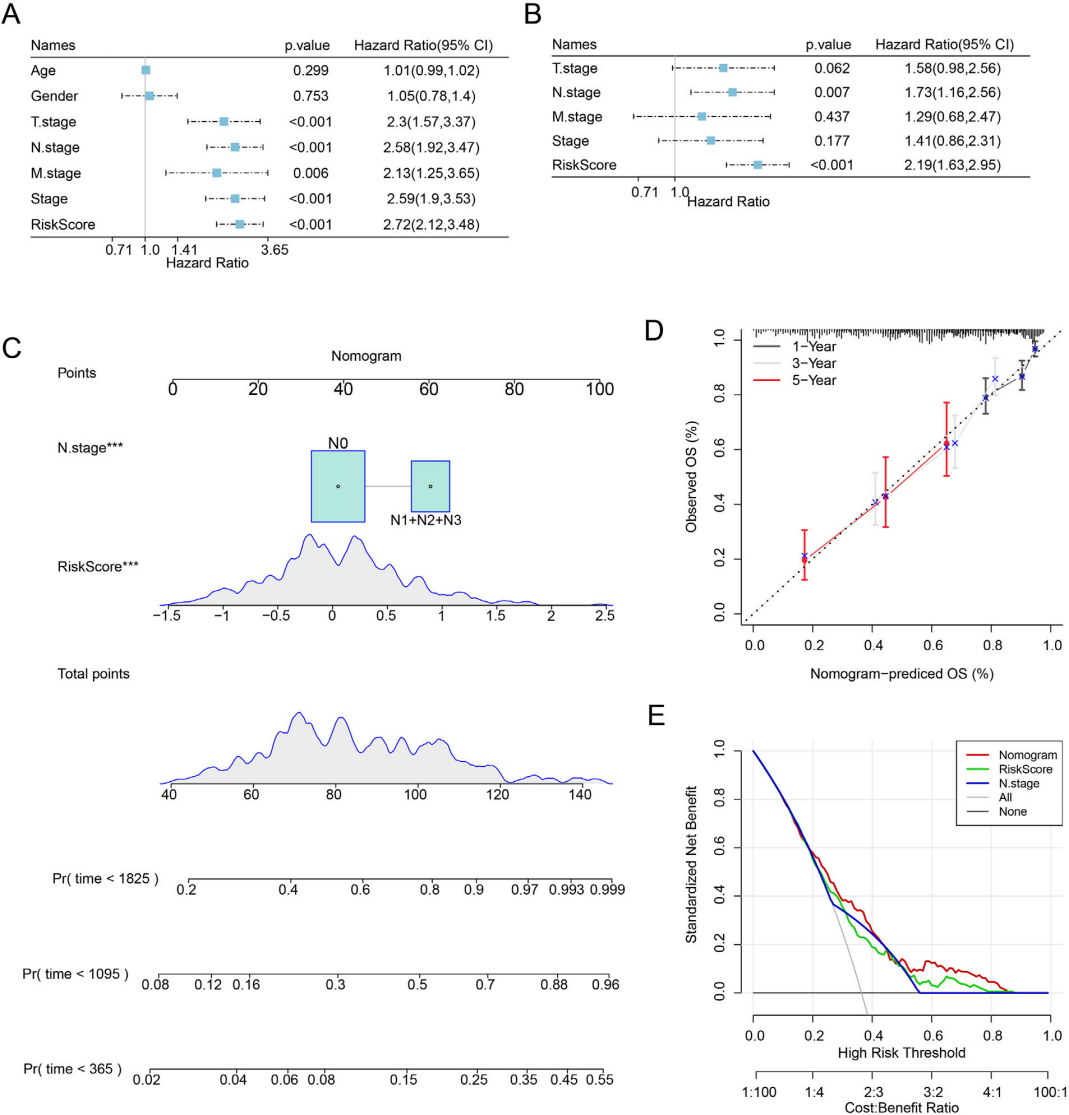
LUAD patients’ prognosis can be predicted via nomogram creation. **(A)** One-way Cox analysis of clinical features and risk score. **(B)** Multifactor Cox analysis of clinical features and risk score. **(C)** Modeling nomograms. **(D,E)** Decision curves and nomogram calibration curves.

First, we assessed the prognosis of LUAD patients by univariate (T.stage: HR (95% CI) = 2.3 (1.57,3.37), *p* < 0.001; N.stage: HR (95% CI) = 2.58 (1.92,3.47), *p* < 0.001; M.stage: HR (95% CI) = 2.13 (1.25,3.65), *p* = 0.006; stage: HR (95% CI) = 2.59 (1.9,3.53), *p* < 0.001; Riskscore: HR (95% CI) = 2.72 (2.12,3.48), *p* < 0.001) and multivariate Cox regression analyses emphasized the importance of the Riskscore (HR (95% CI) = 2.19 (1.63,2.95), *p* < 0.001) and N.stage (HR (95% CI) = 1.73 (1.16,2.56), *p* = 0.007) as significant prognosis factors for LUAD (Figures 4A,B). In order to better quantify the survival probability and risk assessment for LUAD patients, Riskscore with N.stage were combined to construct a nomogram to estimate the OS of LUAD patients at 1-, 3-, and 5-years. Riskscore was the most important factor affecting the OS prediction of LUAD patients, as shown in Figure 4C. Furthermore, we used calibration curves to assess the nomogram model’s prediction accuracy. We found that the 1-, 3-, and 5-year predicted calibration points agreed well with the ideal curves, suggesting that the nomogram had outstanding predictive performance (Figure 4D). We conducted a DCA analysis to assess the model’s dependability, and the results demonstrated that nomogram and Riskscore were the most successful in predicting prognosis, with advantages that were significantly more than those of the baseline model (Figure 4E).

### Differences in immune characteristics among different risk subgroups of LUAD patients

**FIGURE 5:**
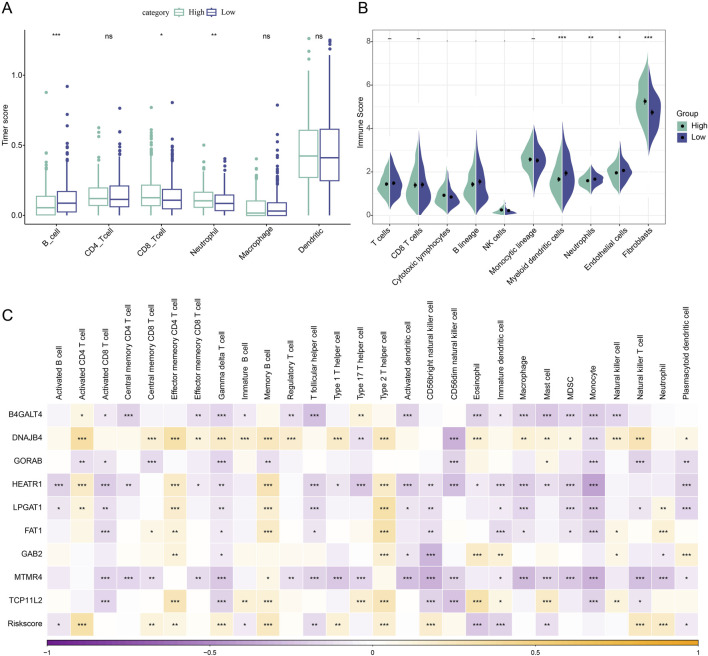
Immunologic characteristics between high and low-risk subgroups of LUAD patients. **(A)** TIMER assessment of immune cell scores in Riskscore subgroup comparisons. **(B)** MCP-counter assessment of immune infiltration in Riskscore subgroup comparison. **(C)** Spearman correlation analysis of 9 biomarkers with 28 immune cell infiltration profiles. *p < 0.05; **p < 0.01; ***p < 0.001.

We first computed the immune cell scores of the TCGA dataset in various subgroups using the TIMER algorithm in order to see the association between Riskscore and immune function in LUAD. The low-risk group’s B_cell score was higher than the high-risk group’s (*p* < 0.001, Figure 5A). The high-risk group had significantly higher CD8_T cell (*p* < 0.05) and neutrophil (*p* < 0.01) scores than the low-risk group. Furthermore, when comparing the high-risk group to the low-risk group, the results of the immune cell score calculation using the MCP algorithm revealed that the immune scores of myeloid dendritic cells (*p* < 0.001), neutrophils (*p* < 0.05), and endothelial cells (*p* < 0.05) were lower in the high-risk group (Figure 5B). The expression of pivotal genes was linked to the infiltration of the great majority of immune cells, according to our calculation of the association between pivotal gene expression profiles and Immune Cell Infiltration Score. In general, *HEATR1* and *MTMR4* genes showed a stronger correlation with immune cell infiltration (Figure 5C).

### Immunotherapy and drug sensitivity assessment of patients in different risk groups of LUAD

**FIGURE 6:**
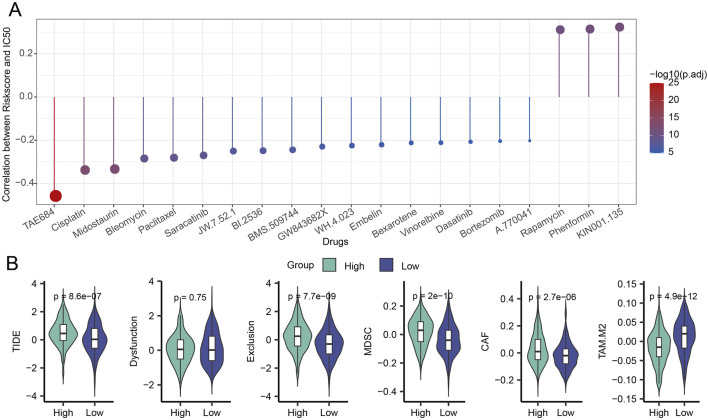
Riskscore, immunotherapy, and medication sensitivity in LUAD are correlated. **(A)** Findings from the association between Riskscore and IC_50_ for drug sensitivity. **(B)** Variation in TIDE analysis outcomes between groups at high and low risk.

To shed light on the relationship between the model and drug sensitivity, we then calculated the Riskscore and IC_50_ correlation to filter for drugs with *p* < 0.05 and predicted chemotherapeutic therapies for patients in the high and low-risk categories of LUAD. Calculations revealed a strong relationship between 21 pharmaceutical sensitivities and Riskscore (Figure 6A). Except for the drugs KIN001.135, Phenformin, and Rapamycin, whose sensitivity had a positive correlation with Riskscore, the other 18 drugs had a negative correlation with Riskscore. TAE684, Cisplatin, and Midostaurin were the three that showed the strongest correlation with Riskscore. The LUAD high-risk group performed better than the LUAD low-risk group in terms of TIDE, myeloid-derived suppressor cells (MDSC), tumor-associated fibroblasts (CAF), and exclusion, as seen in Figure 6B. Here, it is evident that the main way that patients in the high-risk group can evade the immune system is through immunological rejection mechanisms. The low-risk group had higher levels of tumor-associated macrophages of the M2 subtype (TAM.M2) than the high-risk group did, indicating that immune cell suppression may be the primary mechanism by which the low-risk group achieves immunological escape.

### Downregulation of HEATR1 impairs the migratory and invasive capabilities of LUAD cells

**FIGURE 7:**
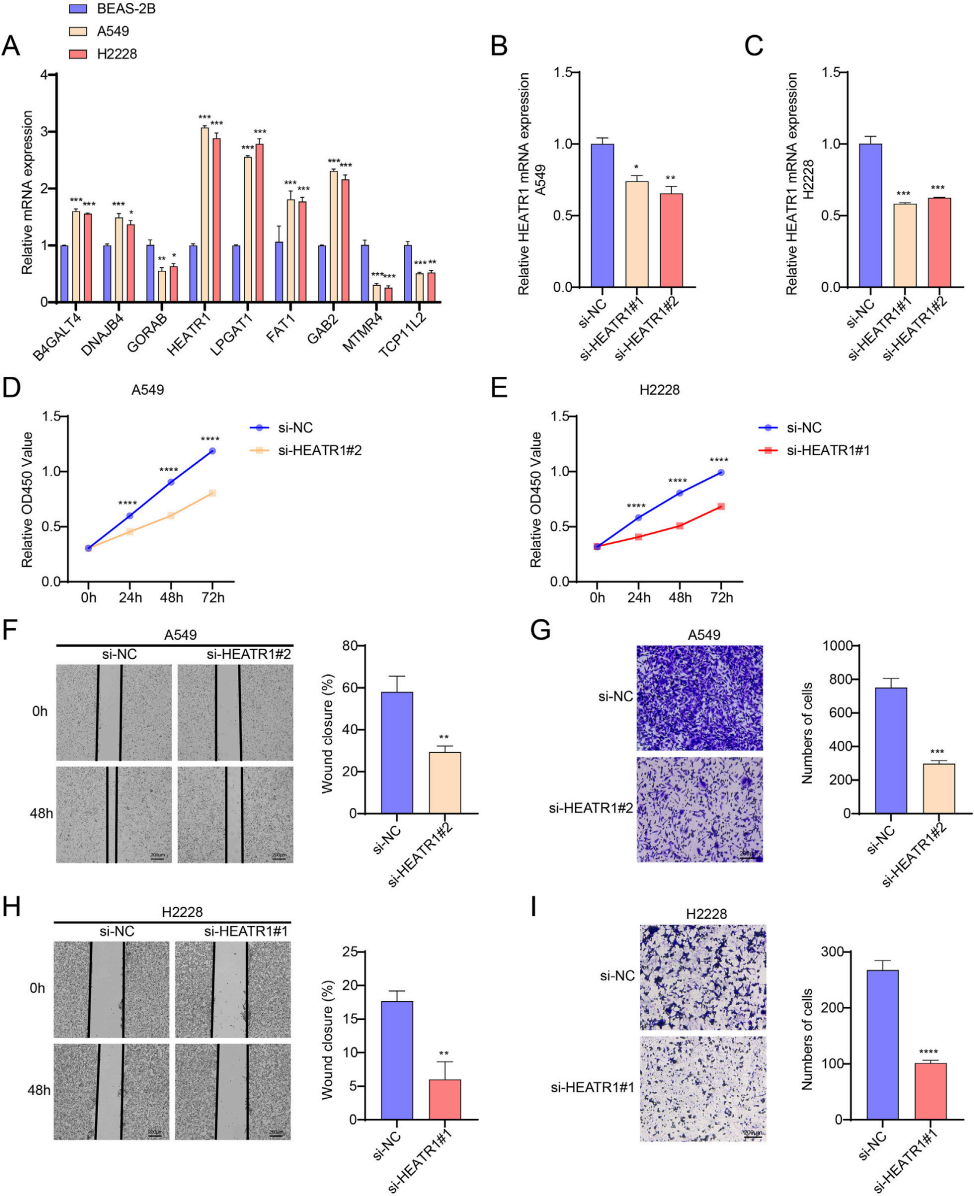
Investigating *HEATR1*’s biological function in LUAD. **(A)** *B4GALT4*, *DNAJB4*, *GORAB*, *HEATR1*, *LPGAT1*, *FAT1*, *GAB2*, *MTMR4* and *TCP11L2* gene expression levels in BEAS-2B, H2228, and A549 cells were detected by qPCR. **(B,C)** Verification of *HEATR1* knockdown’s impact. **(D,E)** Verification of how *HEATR1* knockdown affects H2228 and A549 cell viability. **(F–I)** Representative pictures of H2228 and A549 cells following *HEATR1* knockdown in the wound healing experiment (magnification, ×40; scale bars = 200 μm) and transwell assay (magnification, ×100; scale bars = 200 μm). All procedures were performed in triplicate. SD ± mean, *, *p* < 0.05; **, *p* < 0.01; ***, *p* < 0.001; ****, *p* < 0.0001 are the ways in which the data are presented.

The mRNA expressions of nine important genes in BEAS-2B, H2228, and A549 cells were detected by qPCR. The results showed that both H2228 and A549 cells had considerably lower levels of the *GORAB*, *MTMR4* and *TCP11L2* genes than BEAS-2B cells. In both H2228 and A549 cells, the levels of the genes *B4GALT4*, *DNAJB4*, *HEATR1*, *LPGAT1*, *FAT1* and *GAB2* were markedly elevated (Figure 7A).

We investigated how *HEATR1* contributes to the development of LUAD. Here, we used the CCK8 assay to see how *HEATR1* knockdown affected the viability of H2228 and A549 cells. In order to transfect H2228 and A549 cells, respectively, we first created two *HEATR1* knockdown plasmids. Afterward, we selected the transfected cells with the best knockdown effect (H2228, si-*HEATR1*#1 and A549, si-*HEATR1*#2) for the ensuing tests (Figures 7B,C). *HEATR1* knockdown was shown to dramatically reduce the viability of H2228 and A549 cells (Figures 7D,E). We next used the wound healing assay and the transwell assay to investigate the impact of the *HEATR1* gene on the invasion and metastasis of H2228 and A549 cells. The findings demonstrated that *HEATR1* knockdown markedly reduced H2228 and A549 cell migration and metastasis (Figures 7F–I).

## Discussion

Proteins generated by certain oncogenes may degrade abnormally due to the ubiquitin-associated pathway, which can then cause these proteins to accumulate abnormally in the body. As a result, the ubiquitin system is intimately linked to the initiation and spread of cancer (Reich et al., 2006; Jayadev and Yusuff, 2024). According to recent research, LUAD is associated with downregulated levels of the E3 ubiquitin-protein ligase TRIM3, a crucial regulator of ferroptosis. By raising intracellular ROS and lipid peroxidation levels, high TRIM3 expression encourages cell death (Wang Z. et al., 2024). URGs have been shown to function as immunological traits and tumor progression biomarkers in several tumor types, such as pancreatic cancer (Tan et al., 2024) and hepatocellular carcinoma (Chen et al., 2024). The modular genes in LUAD that had the largest positive connection with URGs were primarily found to be enriched in the pathways of endocytosis, proteoglycans in cancer, and ubiquitin-mediated proteolysis. Zhang et al. demonstrated that endocytosis inhibitors increased apoptosis and suppressed the growth of LUAD cells (Zhang et al., 2023). LUAD cells were more sensitive to gefitinib when the ubiquitin-mediated protein hydrolysis of the epidermal growth factor receptor protein was inhibited (Zhou et al., 2024). Unlike previous studies that focused on single ubiquitination regulators, our study comprehensively integrates URG-based co-expression modules and clinical prognosis to construct a robust predictive model, highlighting novel functional associations between ubiquitin-related genes and LUAD progression.

Nine prognostic-related hub genes were identified by combining common bioinformatics methods from URGs: *B4GALT4*, *DNAJB4*, *GORAB*, *HEATR1*, *LPGAT1*, *FAT1*, *GAB2*, *MTMR4* and *TCP11L2*. The results of the ROC curve study showed that the Riskscore based on the nine hub genes had exceptional diagnostic efficacy for OS in LUAD patients. Glycosyltransferases of the *B4GALT* family are implicated in several biological mechanisms that accelerate the development of cancer. Poor prognosis was tightly linked to microtubule spindle formation and the general upregulation of *B4GALT* transcript levels in hepatocellular cancer tissues (Foote et al., 2023; Dai et al., 2022). Better OS in patients was positively connected with lower levels of *DNAJB4* expression in LUAD tissues (Yu et al., 2020). By blocking MDM2-mediated ubiquitination, lowering cell growth rate, raising apoptosis levels, and reducing tumorigenicity, overexpression of *GORAB* (*SCYL1*-*BP1*) stabilizes functional p53 (Yan et al., 2010). *HEATR1* negatively regulates Akt to increase the cancer cells’ sensitivity to chemotherapy, making it a possible predictive and prognostic biomarker for treatment responsiveness and the prognosis of patients with pancreatic cancer, according to Liu et al. (Liu et al., 2016). *LPGAT1* expression is increased in LUAD tissues. Overexpression of LPGAT1 is associated with a poor outcome in patients with LUAD. *LPGAT1* gene knockdown inhibited the growth and spread of tumors in cellular and animal experiments (Gong et al., 2023). Dendritic cell infiltration is much larger in LUAD patients with higher *FAT1* mutation rates, while memory B cell and resting memory CD4^+^ T cell infiltration is much lower (Feng et al., 2022). *FAT1* mRNA levels are also markedly elevated in LUAD. By controlling TAM.M2 polarization, *GAB2* stimulates the growth of colorectal cancer (Gao et al., 2024). In grade IV glioblastomas, diffuse gliomas exhibit significant expression of *MTMR4* (Bourgonje et al., 2016). Another possible pharmacological target for blocking breast cancer stem cells is *MTMR4* (Hermawan et al., 2024). Through formin-like 2 (*FMNL2*), *TCP11L2*, which is primarily found near microfilaments and microtubules, stimulates the migration and differentiation of satellite cells originating from bovine skeletal muscle (Li S. et al., 2020). According to these findings, biological processes like drug sensitivity, invasion, metastasis, and tumor growth may be linked to URG signature genes. Furthermore, additional *in vivo* and *in vitro* studies are required in the future to examine the biological roles and possible processes of the remaining genes in LUAD, as they have not been described in this condition with the exception of *DNAJB4*, *LPGAT1* and *FAT1*.

Moreover, epidemiological studies have shown that poorer OS in LUAD patients is associated with higher tumor purity, lower immunological scores of the immune microenvironment, and notably lower numbers and immune-related traits of the majority of immune cells (Ma et al., 2022). Therefore, investigating the relationship between tumor immune function and prognosis is instructive for LUAD diagnosis and treatment. According to the current classification, the low-risk group had higher B-cell values. In individuals with LUAD, contact between CD4 T follicular helper cells and B cells was linked to longer life. In a mouse LUAD model, neoantigen-driven B cells and CD4T follicular helper cells work in concert to stimulate anti-tumor immunity and anti-tumor CD8T cell responses (Cui et al., 2021). Furthermore, the high-risk group’s CD8_Tcell and Neutrophi scores were noticeably greater than those of the low-risk group. According to studies, anti-tumor immunity *in vivo* with anti-PD-1 therapy is improved by increased CD8 + T cell activity and abundance in the LUAD tumor microenvironment (Cui et al., 2023). The immunological escape of LUAD cells is mediated by inhibition of CD8^+^ T cell activity (Yang et al., 2024). This could indicate that the high-risk group will respond to immunotherapy at a higher rate. OS and progression-free survival were lower in LUAD patients with a greater neutrophil-to-lymphocyte ratio (Tanizaki et al., 2018). This supports our conclusion that the prognosis is poorer for patients in the high-risk group.

There is a significant influx of cytotoxic T cells in some tumor microenvironments, but these T cells are usually functionally exhausted and no longer able to destroy tumors. On the other hand, immunosuppressive cells (such as CAF, MDSC, and TAM) limit T-cell penetration into the tumor microenvironment (Gajewski et al., 2013; Joyce et al., 2015). Therefore, elucidating the primary immune escape pathways of tumor cells can greatly enhance the efficacy of anti-tumor immunotherapy. The Peng, J. team created an algorithmic framework for TIDE based on genetic elements of T cell dysfunction and T cell exclusion in order to calculate T cell dysfunction and T cell exclusion scores as well as the levels of CAF, MDSC and TAM infiltration in TME (Jiang et al., 2018). Notably, when we compared the potential therapeutic effects of immunotherapy across high and low-risk groups for LUAD using TIDE software, TIDE, CAF, MDSC, and Exclusion scored highest in the high-risk group. According to earlier research, glutamine inflow and a bad prognosis for LUAD patients were linked to exosome expression in CAF (Liu T. et al., 2022). Low CD8^+^ T cell infiltration and poor treatment response of patients to immune-detecting sites were linked to elevated MDSC infiltration in LUAD patients’ tumors (Yin et al., 2022). With anti-programmed cell death protein-1 therapy, inhibition of the immunological rejection-associated neo-oncogene *NME4* in LUAD enhanced CD8^+^ T cell activity and abundance and supported *in vivo* anti-tumor immunity (Zhang et al., 2024). From this, it is clear that immunological rejection mechanisms are the primary means by which patients in the high-risk group may elude the immune system. According to Li et al., LUAD cell invasion, migration, and proliferation were all impeded by TAM.M2 polarization inhibition (Li et al., 2024). On the other hand, the low-risk group’s higher TAM.M2 score suggests that their primary means of immunological escape may be immune cell malfunction.

Additionally, the frequent onset of treatment resistance considerably shortens the survival span of LUAD patients, making therapeutic decisions much more difficult for clinicians (Altorki et al., 2019). Therefore, a comprehensive assessment of the medication sensitivity of LUAD patients is necessary. In this work, we performed a correlation analysis between Riskscore and drug sensitivity. The drugs that had the biggest negative correlation with Riskscore were TAE684, Cisplatin, and Midostaurin; this could help to explain why the high-risk group did not do well. According to earlier research, exogenous EGF in LUAD prevented TAE684 from inhibiting ERK and STAT3 signaling, which reduced the sensitivity of cell growth to TAE684 (Tanizaki et al., 2012). Lowering the expression of E2F7, a crucial gene linked to cisplatin resistance, dramatically decreased LUAD invasion, migration, and proliferation while raising apoptosis (Mao et al., 2024). According to Liu et al., immunological subgroups of LUAD that did not respond well to immunotherapy were more vulnerable to chemotherapy with midostaurin (Liu M. et al., 2022). In conclusion, our research might offer a theoretical foundation for treating LUAD patients on an individual basis.

It is worth noting that our study has certain limitations. For example, this study mainly relies on public datasets for model construction and validation. Although the data quality is high and the sample size is large, due to the heterogeneity of sample collection, sequencing platforms, and processing methods, there may be potential biases that limit the model’s generalization ability in actual clinical scenarios. Future research should integrate data from multi-center, multi-platform, and multi-source clinical samples to further reduce batch effects. Additionally, local clinical cohorts should be collected for prospective validation to enhance the model’s applicability and reliability. In addition, although this study identified nine key genes and validated the function of *HEATR1*, the specific roles of other genes in LUAD and their potential regulatory mechanisms have not been thoroughly explored, nor has it been clarified whether there is a synergistic network among them. In the future, we will conduct more *in vitro* and *in vivo* experiments, such as gene overexpression/knockout and mouse xenograft models, to systematically validate their roles in LUAD and explore the regulatory relationships and signaling axes among key genes, thereby revealing potential synergistic mechanisms. Finally, although we have constructed a prognostic model, it has not yet been prospectively validated in real-world clinical practice, nor has its practical guidance value for individualized treatment strategies been explored. Therefore, future studies should conduct independent validation in prospective clinical cohorts to assess the model’s actual efficacy in predicting prognosis, immune therapy response, or chemotherapy response in LUAD patients. Additionally, it should be integrated and compared with existing clinical scoring systems to explore its supplementary value in clinical decision-making.

## Conclusion

In order to create a prognostic model with high robustness, we employed machine learning techniques to screen three modular genes that had the strongest correlation with URGs. We then took the intersection of these genes with DEGs between LUAD samples and control samples and screened nine featured genes. It can accurately forecast LUAD patients’ prognosis, immunotherapy, immunological infiltration, and sensitivity to anticancer medications, giving doctors a solid foundation on which to build individualized treatment plans. This study can serve as a theoretical guide for future investigations into possible biomarkers for LUAD patient diagnosis and prognosis prediction.

## Data Availability

The datasets presented in this study can be found in online repositories. The names of the repository/repositories and accession number(s) can be found in the article/supplementary material.

## Abbreviations

LUAD, Lung adenocarcinoma; URGs, Ubiquitin-related genes; WGCNA, Weighted gene co-expression network analysis; GO, Gene ontology; KEGG, Kyoto Encyclopedia of Genes and Genomes; BP, Biological process; MF, Molecular function; CC, Cellular component; TAM.M2, Tumor-associated macrophages of the M2 subtype; MDSC, Myeloid-derived suppressor cells; CAF, Tumor-associated fibroblasts; DEGs, Differentially expressed genes; KM, Kaplan-Meier; GDSC, Drug sensitivity in cancer; IC50, Inhibitory concentration; AUC, Area under ROC curve; DCA, Decision curve analysis; GEO, Gene expression omnibus; LASSO, Least absolute shrinkage and selection operator; OS, Overall survival; ROC, Receiver operating characteristic analysis; GSVA, Gene set variant analysis; ssGSEA, single-sample gene set enrichment analysis; TCGA, The cancer genome atlas; TIDE, Tumor immune dysfunction and exclusion.
